# Clinical value of FNA puncture feeling in the diagnosis of non-diagnostic and indeterminate thyroid nodules

**DOI:** 10.3389/fendo.2022.1022438

**Published:** 2022-11-02

**Authors:** Jintao Wu, Yingying Li, Mingbo Zhang

**Affiliations:** ^1^ Department of Ultrasound, Linzi District Maternity and Child Health Care Hospital, Zibo, China; ^2^ Department of Ultrasound, Chinese PLA General Hospital, Beijing, China

**Keywords:** Fine needle aspiration (FNA), thyroid nodules, thyroid cancer, puncture feeling, ultrasound

## Abstract

**Objective:**

The aim of this study was to explore the clinical diagnostic value of puncture feeling during fine-needle aspiration (FNA) for non-diagnostic and indeterminate thyroid nodules.

**Methods:**

A retrospective analysis was performed on 176 patients (196 nodules) diagnosed with Bethesda I or III by FNA cytology at the ultrasound department of our hospital between January 2017 and January 2020. Comparisons were made on the differences in puncture feeling (including stiffness and grittiness) between benign and malignant thyroid nodules, and their diagnostic performance was analyzed.

**Results:**

There were significant differences between benign and malignant nodules with respect to the puncture stiffness and puncture grittiness (*P* < 0.001). The presence of a hard stiffness and grittiness demonstrated comparable levels of diagnostic performance for malignant thyroid nodules, with sensitivities, specificities, accuracies, positive predictive values, and negative predicative values of 55.56% and 63.89%, 87.10% and 78.22%, 75.51% and 72.96%, 71.43% and 63.01%, and 77.14% and 78.86%, respectively. The diagnostic performance was most optimal in the presence of at least one puncture feeling (area under the receiver operating characteristic curve: 0.771), exhibiting a sensitivity, specificity, accuracy, positive predictive value, and negative predictive value of 79.17%, 75.00%, 76.53%, 64.77, and 86.11%%, respectively.

**Conclusions:**

Puncture feeling adds clinical value in the diagnosis of thyroid nodules with indeterminate FNA findings.

## Introduction

There has been a significantly gradual rise in the incidence of thyroid cancer over the past few years (1). Fine-needle aspiration (FNA) is a cytopathological technique that plays a crucial role in the diagnosis of thyroid cancer (2, 3). The diagnosis through FNA cytology is reliant on the Bethesda System for Reporting Thyroid Cytopathology (4). However, there are ambiguities in its criteria for cytological diagnosis, and 10-15% false negatives has been reported (5, 6). In particular, Bethesda category I and III nodules are non-diagnostic and indeterminate thyroid nodules prone to misdiagnosis, which inevitably cause repeat aspiration or diagnostic operation.

Molecular pathology including gene mutational panel and comprehensive multigene next-generation sequencing panel can be used to diagnosis thyroid cancer (7). However, the cost is expensive and not universally available. Repeat FNA of non-diagnostic and indeterminate thyroid nodules had a positive impact. However, some cases may remain indeterminate even after repeat aspiration of the nodule (8).During the process of FNA, the operator may detect different sensations as the needle enters the nodule (i.e., puncture feeling), such as stiffness, grittiness, etc. The different sensations indicate different internal composition of the nodules, which may contribute to differential diagnosis. Due to the presence of calcifications and psammoma bodies in papillary thyroid carcinomas, the friction may contribute to the sensation of grittiness during the puncture process (9). In addition, most thyroid cancers appear as hard nodules on elastography (10). At present, research on the significance of puncture feeling to the diagnostic performance of thyroid nodule biopsy is relatively scarce, and it remains unclear which types of puncture feeling can better facilitate the diagnosis of thyroid nodules.

In order to further improve diagnostic accuracy on the basis of cytopathological diagnosis and to provide evidence for decision-making by clinicians and interventional sonographers, this study explored the clinical diagnostic value of puncture feeling during FNA biopsy for non-diagnostic and indeterminate nodules and compared the diagnostic performances of different commonly observed puncture feelings.

## Materials and methods

### Patients

176 patients (196 nodules) diagnosed with Bethesda category I or III nodules by thyroid nodule FNA cytology at the ultrasound department of Qidu Hospital between January 2017 and January 2020 were enrolled in this retrospective study.

The inclusion criteria were as follows (1): patients with well-defined single or multiple thyroid nodules that were suspicious for malignancy and indicated for FNA biopsy; (2) patients who underwent ultrasound-guided FNA at our department; (3) cytopathological findings indicating Bethesda category I or III nodules; (4) patients who underwent repeat aspiration or surgical treatment after FNA. The exclusion criteria: patients with unclear secondary aspiration or postoperative pathological findings.

### Instruments and methods

Thyroid examination was performed using the Mindray Resona 7 (Mindray, Shenzhen, Guangdong, China) color Doppler ultrasound system equipped with an L14-5 probe, and the settings were adjusted for thyroid examination (frequency: 5–14 MHz). In accordance with the classification criteria of the American College of Radiology (ACR) Thyroid Imaging, Reporting and Data System (TI-RADS) (2), the thyroid nodules that met the ACR criteria or 2012 Chinese Medical Association Guidelines (11) were recommended for FNA. Prior to the biopsy, informed consent was obtained in writing from the patients and their family members. FNA was performed using 25G × 50 mm biopsy needles (Hakko Co., Ltd., Chikum-Shi, Nagano, Japan).

All FNA procedures were performed by a sonographer with more than 10 years of interventional experience. During the procedure, patients were placed in a supine position, and the puncture site was covered with fenestrated drapes after undergoing disinfection. The sonographer sat at the patient’s cranial end. Using the freehand FNA technique, the needle was inserted at the midpoint on the side of the probe. Under ultrasound guidance, the needle tip entered the nodule without negative pressure, and 10–20 needle passes were made in multiple directions. The assistant observed whether bleeding occurred at the end of the needle and prompted the sonographer to stop if bleeding occurred to prevent excessive blood in the biopsy specimen. The puncture feeling was dictated and documented by the operator. Grittiness was defined as a sensation of sandpaper-like friction during the needle insertion, and its presence or absence was recorded. In addition, the stiffness encountered when the needle tip entered the nodule was compared with that entering normal thyroid parenchyma. Nodules with a higher stiffness than normal thyroid parenchyma were defined as hard. Otherwise, they were defined as soft. Once FNA was completed, rapid smear preparation was performed with the cooperation of the assistant, and the specimens were fixed using 95% alcohol for 20 s. The operator then selected 2–3 smears based on visual judgement of the specimen quality for cytopathological examination in the pathology department. The pathological findings were classified according to the Bethesda System for Reporting Thyroid Cytopathology (12). Patients with nodules classified as category I or category III were recommended for repeat aspiration or surgical treatment, as appropriate.

The medical records of patients with thyroid nodules stored in the ultrasound department were reviewed. Screening was performed using the clinical data and pathological records to select the patients with Bethesda category I or III nodules. The patients’ parameters were recorded, which included sex, age, maximum nodule diameter, nodule volume (superior-inferior diameter × left-right diameter × anterior-posterior diameter × 0.52), initial FNA findings and final pathological findings. Comparisons were performed to determine whether the presence/absence of grittiness and nodule puncture texture was associated with the final pathological findings (benign vs. malignant). In addition, we evaluated the clinical value of applying the two types of puncture feelings alone or combined in the diagnosis of thyroid nodules.

### Statistical methods

All statistical analyses were performed using SPSS (version 26.0). Measurement data are expressed as the mean ± standard deviation, whereas the count data are expressed as the number of cases and percentage. Comparisons of differences were performed using the chi-squared test or Fisher’s exact test. The diagnostic performance of puncture feeling for thyroid nodules was evaluated using its sensitivity, specificity, accuracy, positive predictive value (PPV), negative predictive value (NPV), receiver operating characteristic (ROC) curve and area under the ROC curve (AUROC). *P* < 0.05 was used to indicate statistical significance.

## Results

### Clinical and pathological data

A total of 176 patients with 196 nodules were enrolled, including 21 males (11.9%) and 155 females (88.1%), and the patients had a mean age of 43.48 ± 11.39 years (20–72 years). The maximum nodule diameter was 2.42 ± 1.59 cm (0.5–6.6 cm), and the nodule volume was 7.78 ± 2.01 (0.065–66.25 cm^3^). The initial FNA findings indicated that 115 (58.7%) were Bethesda category III nodules and 81(41.3%) were category I nodules. The final pathological confirmation indicated that 124 were benign nodules, including 61 (49.2%) nodular goiter, 5 (4.0%) subacute thyroiditis, 40 (32.3%) thyroid adenoma and 18 (14.5%) localized Hashimoto’s thyroiditis; whereas 72 were malignant nodules, including 70 (97.2%) papillary thyroid carcinoma and 2 (2.8%) follicular thyroid carcinoma. Of all the nodules enrolled in our study, their tumor size, location and ultrasound characteristics were displayed in **Table 1**.

**Table 1:** Imaging features of the thyroid nodules.

Parameter	Result	Pathology	
		Malignant	Benign
Diameter (cm)		1.49±1.08	2.95±1.59
Location	Left lobe	30 (41.67)	57 (45.97)
	Right lobe	35 (48.61)	58 (46.77)
	Isthmus	7 (9.72)	9 (7.26)
echogenicity	Hypoechogenicity	59 (81.94)	75 (60.48)
	Isoechogenicity	2 (2.78)	4 (3.23)
	Hyperechogenicity	1 (1.39)	2 (1.61)
	Mixed echogenicity	10 (13.89)	43 (34.68)
Composition	Mixed cystic and solid	11 (15.28)	37 (29.84)
	Solid or almost solid	61 (84.72)	87 (70.16)
Shape	Wider-than-tall	40 (55.56)	109 (87.90)
	Taller-than-wide	32 (44.44)	15 (12.10)
Echogenic foci	Microcalcification	14 (19.44)	21 (16.94)
	Macrocalcification	29 (40.28)	23 (18.55)
	Peripheral calcification	19 (26.39)	4 (3.23)
	None	10 (13.89)	76 (61.29)
Vascular pattern	intratumoral	22 (30.56)	79 (63.71)
	Periphtumoral	37 (51.39)	14 (11.29)
	Both	13 (18.06)	31 (25.00)
Total		72	124

Values are presented as number and percentage (parenthesis).

Of all the 72 malignant nodules, 9 of them (12.5%) were presence with psamoma on histology. And tumor variant results showed 2(2.8%) medullary carcinoma, 3(4.2%) follicular papillary carcinoma, 15(20.8%) micro-carcinoma and 52(72.2%) classical papillary carcinoma. 64(88.9%) received surgery and 8(11.1%) received ultrasound guided thermal ablation. Of the 64 patients who underwent surgery, the results of the T-staging showed 59(92.2%) T1, three (4.7%) T2 and two (3.1%) T4. Their N-staging showed 27(42.2%) N1 and 37(57.8%) N0. Occult cancers were detected in 12(18.8%) cases. The 8 patients who underwent ablation were all confirmed as classical papillary carcinoma by CNB before ablation, and the ablation was successfully completed, with more than 1 year’s follow-up. No tumor recurrence and lymph node metastasis were detected in the 8 ablation cases.

### Diagnostic performance of puncture feeling in thyroid nodules with indeterminate FNA findings

As shown in **Table 2**, there were significant differences between benign and malignant nodules with respect to the nodule texture (hard vs. soft) (*P* < 0.001) and presence of grittiness (*P* < 0.001) during the puncture.

**Table 2:** Differences in the puncture feeling of thyroid nodules with indeterminate FNA findings.

Parameter	Result	Pathology		χ^2^	*P* value
		Malignant	Benign		
Puncture texture	Hard	40	16	40.61	<0.001
	Soft	32	108		
Grittiness	Present	46	27	34.57	<0.001
	Absent	26	97		
Number of puncture feelings	Both	29	12	25.78	<0.001
	Either	28	19	13.88	<0.001
	At least one	57	31	54.02	<0.001
	None	15	93	54.02	<0.001

As shown in **Table 3**, the puncture stiffness exhibited a diagnostic sensitivity of 55.56%, specificity of 87.10%, accuracy of 75.51%, PPV of 71.43%, and NPV of 77.14% in the diagnosis of benign and malignant thyroid nodules; while the puncture grittiness exhibited a diagnostic sensitivity of 63.89%, specificity of 78.22%, accuracy of 72.96%, PPV of 63.01%, and NPV of 78.86% in the differential diagnosis of benign and malignant thyroid nodules.

**Table 3:** Diagnostic performance of puncture feeling for thyroid nodules with indeterminate FNA findings.

Parameter	Sensitivity	Specificity	Accuracy	PPV	NPV	AUROC	95%CI
Stiffness	55.56%	87.10%	75.51%	71.43%	77.14%	0.713±0.040	0.634~0.793
Grittiness	63.89%	78.22%	72.96%	63.01%	78.86%	0.711±0.040	0.633~0.788
Presence of both	40.28%	90.32%	71.94%	70.73%	72.26%	0.653±0.043	0.569~0.737
Presence of either	38.89%	84.68%	67.86%	59.57%	70.47%	0.618±0.043	0.534~0.702
At least one feeling	79.17%	75.00%	76.53%	64.77%	86.11%	0.771±0.036	0.701~0.841
Absence of both	20.83%	25.00%	13.89%	35.23%	23.47%	0.229±0.036	0.159~0.299

PPV, positive predictive value; NPV, negative predictive value; AUROC, area under the receiver operating characteristic curve; CI, confidential interval.

**Figure 1:**
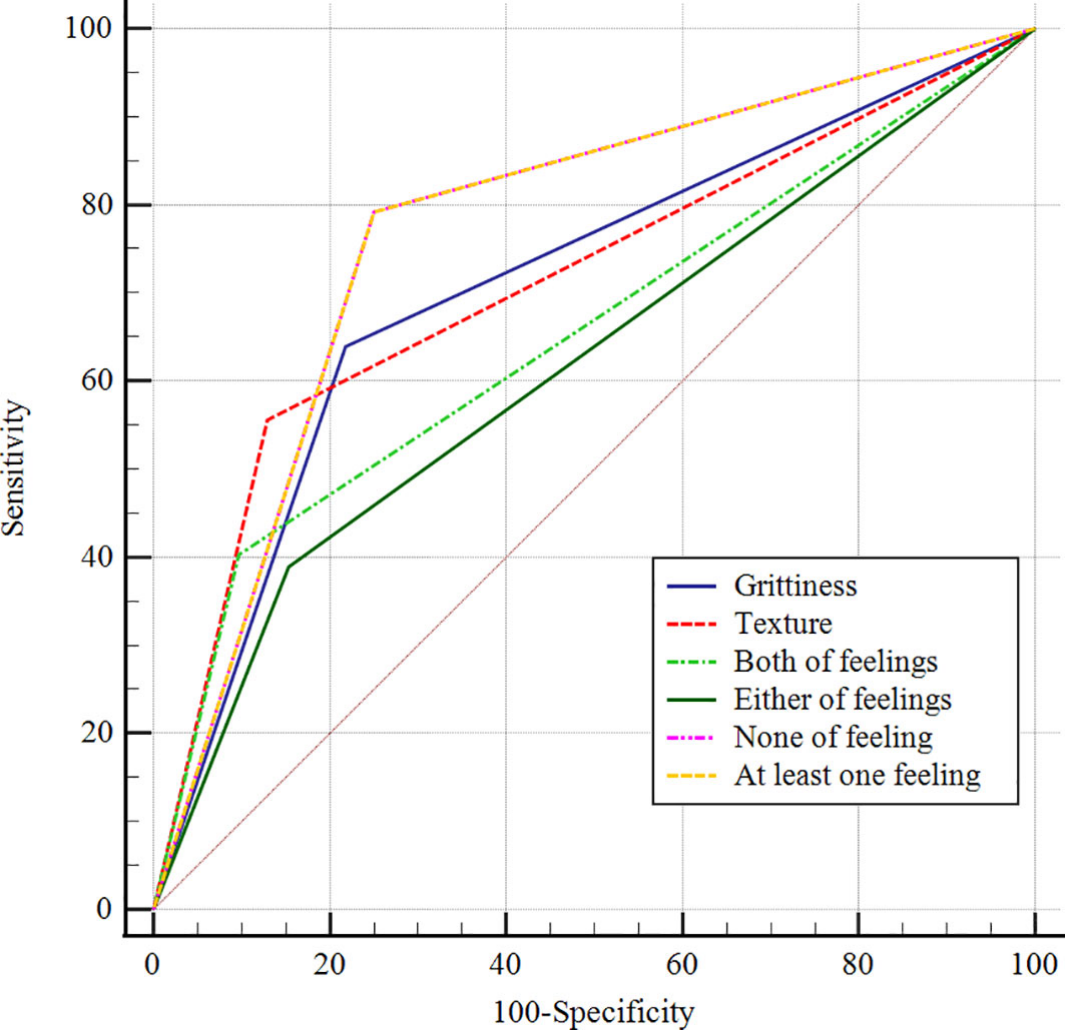
ROC curves for puncture feeling in the differential diagnosis of benign and malignant thyroid nodules with indeterminate FNA findings.

In the differential diagnosis of benign and malignant thyroid nodules with indeterminate FNA findings, the presence of stiffness produced an AUROC of 0.713, while the presence of grittiness produced an AUROC of 0.711. When at least one of the both feelings were present, we can get the highest sensitivity, accuracy, NPC and AUROC of 0.778, 0.811, 0.853 and 0.764; when both hard texture and grittiness were present, the AUROC was 0.653 with the highest specificity of 90.32%. The diagnostic ROC curves are shown in **Figure 1**.

Due to the inflammatory processes can affect the feeling of stiffness and grittiness, we perform an analysis between the inflammatory lesion and malignant thyroid nodules. The puncture stiffness exhibited a diagnostic sensitivity of 55.56%, specificity of 86.96%, accuracy of 63.16%, PPV of 93.02%, and NPV of 38.46%; while the puncture grittiness exhibited a diagnostic sensitivity of 63.89%, specificity of 78.26%, accuracy of 67.37%, PPV of 90.20%, and NPV of 41.86% in the differential diagnosis of inflammatory and malignant thyroid nodules.

## Discussion

FNA is one of the most reliable techniques for the diagnosis of thyroid malignancies and selection of treatment methods and has been recommended by numerous guidelines published in China and abroad (2, 13, 14). However, due to the ambiguities in the FNA findings, nodules with indeterminate pathological reports will often require patients to undergo repeat biopsy. In this study, we demonstrated that puncture feeling had some value in assisting the diagnosis of thyroid nodules with indeterminate pathological findings. Thus, reporting the puncture feeling can provide clinicians with auxiliary diagnostic evidence in cases with indeterminate pathological diagnosis.

Our findings revealed that the sensations of nodule texture perceived during puncture can serve as an auxiliary diagnostic method. Previous studies have demonstrated that malignant tumors exhibited a loss of normal orderly arrangement and an increase in density (15). Furthermore, the presence of proliferative stromal fibrosis in thyroid cancer tissues contributes to tumor hardening. In addition, the presence of calcifications, especially macrocalcifications, in malignant thyroid tumors can also increase their stiffness. Studies using ultrasound elastography have demonstrated that malignant thyroid tumors exhibit a significant higher stiffness than benign tumors (16). However, sonoelastography is more operator-dependent and may be subjected to some interfering factors (17). Moreover, the instrumentation requirements of this technique are not conducive to its widespread promotion in primary care settings. Despite the lack of an objective indicator for evaluating nodule stiffness in this study, the use of puncture feeling to determine the nodule stiffness was more direct. Therefore, this indicator can exclude patients with benign tumors more effectively, which in favor of reducing unnecessary repeat aspirations and diagnostic operation.

Our findings also indicate that puncture grittiness has clinical significance in aiding the diagnosis. The feeling of grittiness encountered during puncture may result from the friction between the nodule calcifications and the needle tip, while nodule microcalcifications are one of the distinct manifestations of malignant thyroid tumors, especially papillary thyroid carcinomas (18). According to histopathological studies, microcalcifications chiefly manifest as psammoma bodies. Psammoma bodies are currently defined as round concentric lamellar calcifications observed in the stroma of papillary carcinomas, and the exact mechanisms underlying their pathogenesis are poorly understood (19). However, they are detected in more than 50% of all papillary thyroid carcinomas (20). Owing to its unique nature, the puncture feeling of grittiness obtained good agreement among experienced operators (8), which also served as a basis for its reliability. In addition, compared with puncture texture, grittiness demonstrated a higher sensitivity, which enabled the more-effective screening of suspicious patients, thereby reducing a missed diagnosis in cases of an indeterminate pathological diagnosis.

Furthermore, our findings suggest that, for nodules with heterogenous texture, the lesion sites were often located in areas with hard texture and grittiness. Hence, focusing the biopsy to such areas can also improve the accuracy of FNA. In addition, providing timely remarks on the puncture feeling or suspicions in ultrasound diagnosis can also facilitate a targeted cytological diagnosis by pathologists, thereby reducing the risk of misdiagnosis.

The diagnostic performance of combining the two types of puncture feelings was also validated in this study. In the presence of both puncture feelings, the diagnostic specificity was 90.32%, which essentially reduced erroneous diagnosis of thyroid cancer, and reduced the pain and medical expenses caused by repeat aspiration. In the presence of at least one puncture feeling, the sensitivity was 79.17%, which reduced misdiagnosis and can serve as an indication for clinicians to arrange for a repeat aspiration promptly or switch to histological biopsy. Li has reported FNA combined with puncture feeling in the diagnosis of thyroid nodules (21). The result showed that the puncture feeling had a good PPV and NPV, which was similar to our result. However, their study included nodules with all Bethesda categories. 74% of them were classified as category V and VI and only 14% of them were classified as category I and III. In contrast, only non-diagnostic and indeterminate thyroid nodules were included in our study. Therefore, the results of our study are more valuable for the diagnosis of such nodules.

There are several limitations to this study. First, the subjectivity of the evaluation indicator (puncture feeling) may lead to significant disparities among different operators. We did not evaluate the concordance among physicians with different puncture experiences, which can be further explored in subsequent studies. Second, the puncture feeling produced in this study cannot be confirmed using objective indicators at present. Finally, this was a single-center retrospective study with a relatively small sample size, and it also did not include rare malignancies (e.g., undifferentiated carcinoma, metastatic carcinoma, etc.). Thus, the sample size should be further increased and prospective studies should be performed in the future.

In conclusion, puncture feeling during FNA may assist the diagnosis of thyroid nodules with indeterminate cytopathological findings, thereby providing the basis for reducing unnecessary repeat aspirations or preventing missed diagnoses. Hence, it has potential value in clinical application.

## Data availability statement

The raw data supporting the conclusions of this article will be made available by the authors, without undue reservation.

## Ethics statement

The studies involving human participants were reviewed and approved by Institutional Ethics Committee of the Linzi Maternal and Child Health Care Hospital (Qidu Hospital). The patients/participants provided their written informed consent to participate in this study.

## Author contributions

All authors listed have made a substantial, direct, and intellectual contribution to the work and approved it for publication.

## Acknowledgments

We are grateful to the patients who participated in this study.

## Conflict of interest

The authors declare that the research was conducted in the absence of any commercial or financial relationships that could be construed as a potential conflict of interest.

## Publisher’s note

All claims expressed in this article are solely those of the authors and do not necessarily represent those of their affiliated organizations, or those of the publisher, the editors and the reviewers. Any product that may be evaluated in this article, or claim that may be made by its manufacturer, is not guaranteed or endorsed by the publisher.
